# Optimal catheter ablation procedure for premature ventricular contraction originating from the free wall of tricuspid annulus

**DOI:** 10.1002/clc.24225

**Published:** 2024-02-02

**Authors:** Naoya Kataoka, Teruhiko Imamura

**Affiliations:** ^1^ Second Department of Internal Medicine University of Toyama Toyama Japan

To the Editor,

1

Chen et al. have illustrated the superior feasibility and efficacy of a novel reserved S‐curve technique (RST), facilitated by a steerable sheath, in the ablation of premature ventricular contractions (PVC) originating from the free wall of the tricuspid annulus, in comparison to the conventional reserved C‐curve technique (RCT).^1^

To determine the earliest activation site, the authors employed systematic mapping with an ablation catheter.^1^ The feasibility of the Octaray catheter (Biosense Webster) for CARTO mapping and the HD grid (Abbott) for EnSite mapping has been well‐established.^2^, ^3^ It is pertinent for the authors to deliberate on the potential causal relationship between the choice of mapping catheter and the occurrence of procedure‐related complications. Two patients in the RCT group experienced cardiac tamponade, underscoring the importance of this discussion.

The matter of anesthesia remains unclear, with the authors not specifying whether general anesthesia or local anesthesia was employed.^1^ General anesthesia may suppress the incidence of PVC and has the potential to be linked with procedural failure. Conversely, it can effectively manage the patient's respiratory status. Given the pronounced influence of respiratory motion on the free wall of the right ventricle, where catheter ablation is associated with a heightened risk of cardiac tamponade, the authors should provide insights into their choice of anesthesia and its implications on the procedure.

Spatial displacement plays a pivotal role in the success of PVC catheter ablation. The free wall of the right ventricle represents a region with substantial spatial displacement. Consequently, the earliest activation site of PVC visualized in three‐dimensional space obtained during sinus rhythm may yield different results from the precise site, and this variance might contribute to the higher success rate observed in the RST group.^1^ Additionally, the CARTO3 system presents an innovative approach for adjusting spatial displacement, which should be explored further.

Ablation time is another crucial aspect deserving consideration. In their study, ablation durations ranged from 90 to 120 seconds at 35 W.^1^ Recent advances propose significantly shorter ablation times, such as 4 seconds at 90 W, to mitigate procedure‐related complications while preserving efficacy.^4^ The authors should elucidate their rationale for the selected ablation time and consider the optimal duration in light of these advancements.

## CONFLICT OF INTEREST STATEMENT

The authors declare no conflict of interest.

## Data Availability

Data sharing is not applicable to this article as no datasets were generated or analyzed during the current study.
